# Paying people to eat or not to eat? Carryover effects of monetary incentives on eating behaviour

**DOI:** 10.1016/j.socscimed.2015.04.002

**Published:** 2015-05

**Authors:** Paul Dolan, Matteo M. Galizzi, Daniel Navarro-Martinez

**Affiliations:** a London School of Economics and Political Science, Department of Social Policy, LSE Health, Houghton Street, WC2A2AE London, UK; b Centre for the Study of Incentives in Health, Institute of Psychiatry, King's College London, Guy's Hospital, St Thomas Street, SE1 9RT London, UK; c Paris School of Economics-École d'Économie de Paris, Hospinnomics, Hôtel-Dieu, 1, Parvis de Notre-Dame, Bâtiment B1, 5° étage, 75004 Paris, France; d Universitat Pompeu Fabra, Departament d'Economia i Empresa, Barcelona Graduate School of Economics, and Barcelona School of Management, Ramon Trias Fargas 25-27, 08005 Barcelona, Spain

**Keywords:** United Kingdom, Monetary incentives, Eating behaviour, Carryover effects, Crowding out of intrinsic motivation, Obesity, Experimental economics, Behavioural economics

## Abstract

There is no evidence comparing head-to-head the effects of monetary incentives to act and to abstain from acting on behaviour. We present an experiment, conducted between June and September 2012, that directly compares the effects of those two different monetary incentive schemes on eating behaviour: we evaluate incentives to eat against incentives not to eat. A large number of participants (n = 353) had bowls of sweets next to them while they watched different videos over two experimental sessions that were two days apart. Sweets eating was monitored and monetary incentives to eat or not to eat were introduced during one of the videos for participants randomly allocated to these conditions. Our results show that, while both types of incentives were effective in changing sweets-eating behaviour when they were in place, only incentives not to eat had significant carryover effects after they were removed. Those effects were still significant two days after the monetary incentives had been eliminated. We also present some additional results on personality and health-related variables that shed further light on these effects. Overall, our study shows that incentives not to eat can be more effective in producing carryover effects on behaviour in domains like the one explored here.

## Highlights

• We experimentally compare the effects of different incentives on eating behaviour.
• Subjects had bowls of sweets next to them while they watched videos in lab sessions.
• Incentives to eat or not to eat were randomly allocated during one of the videos.
• Both incentives were effective in changing eating behaviour when they were in place.
• Only incentives not to eat had significant carryover effects after being removed.

## 1 Introduction

The use of incentives to motivate people lies at the heart of economics (Smith, 1776; Barnard, 1938; Camerer and Hogarth, 1999; Laffont and Martimort, 2002). Recently, financial incentives have been used in a variety of research and policy contexts to induce behaviour change in health-related settings, such as smoking cessation (Volpp et al., 2006, 2009), dieting (Volpp et al., 2008; John et al., 2011; John et al., 2012; Kullgren et al., 2013), exercising (Charness and Gneezy, 2009), and the consumption of fruit and vegetables (Cooke et al., 2011). These studies have typically found that monetary incentives are able to induce significant changes in health behaviour, at least in the short run (Marteau et al., 2009; Gneezy et al., 2011; Volpp et al., 2011; Galizzi, 2014).

Behavioural research, however, has also uncovered a series of effects and principles that are more complex than the mere impact on the targeted behaviour. Financial incentives, in particular, have been associated to unintended effects and ‘hidden costs’ (Fehr and List, 2004) such as the crowding out of intrinsic motivation (Frey and Oberholzer-Gee, 1997; Deci et al., 1999; Fehr and Falk, 2002); changes in social norms or individual beliefs about social norms (Gneezy and Rustichini, 2000a,b; Heyman and Ariely, 2004); the interaction with reciprocity (Fehr and Gachter, 1997; Rigdon, 2009; Dur et al., 2010); reputational concerns (Benabou and Tirole, 2006; Ariely et al., 2009a); or social comparison (Gachter and Thoni, 2010; Greiner et al., 2011).

Studies have also started exploring the unintended ‘spillover’ effects of incentives on behaviours other than the ones directly targeted (Wisdom et al., 2010; Dolan and Galizzi, 2014, 2015), or the conditions under which they adversely lead to ‘choking under pressure’ (Ariely et al., 2009b). To complicate things further, existing studies have examined either incentives to act or to abstain from acting in certain ways, but not both of them together in the same study. This makes it difficult to compare systematically the consequences of these different incentive schemes, including what happens when they are removed.

Such a comparison is of key interest for health policy purposes, as in real world applications incentives will often need to be removed at some point, and both paying to act and paying to abstain from acting could have potential backfire effects once removed. For example, we could pay people not to eat fat foods for some time and then remove the incentive. This could result in reduced calorie intake if the intervention helps people build up healthier eating habits, but, based on what we know about motivation, it could also potentially increase the consumption of calorie dense foods if it undermines people's intrinsic motivation to control their eating in the absence of incentives. An alternative option would be, for instance, to pay people to eat low-fat food items and then remove the incentive. The precise results of these alternative interventions would depend on many factors, but in order to be able to compare directly the merits of incentives to act and to abstain from acting, we need a clean comparison using exactly the same target behaviour and environment.

To the best of our knowledge, this paper presents the first controlled head-to-head study of the effects of monetary incentives to act and to abstain from acting on behaviour. We focus on eating behaviour, which is an issue of significant health policy relevance, and which has already received attention in previous studies of incentives (Jeffery et al., 1993; Cooke et al., 2011; Grubliauskiene et al., 2012; Remington et al., 2012; Wengreen et al., 2013). In particular, we look at sweets eating because it is an ambivalent, stylised health-related behaviour: while eating sweets is a pleasurable, tempting activity, it may be potentially harmful, and even unwanted at a meta level. Many other risky health behaviours, such as alcohol drinking and unsafe sex, share this same common feature of being ambivalent activities. Incentives for sweets eating, moreover, can be readily manipulated in the lab.

We conducted a laboratory experiment in which participants had bowls of sweets next to them while they watched different videos over two sessions set two days apart. During the first session we introduced monetary incentives to eat or not to eat sweets from the bowl and monitored how that affected eating behaviour while the incentives were in place, after they were removed on the same day, and two days after they were removed.

The rest of the paper is organised as follows: Section 2 describes the method used; Section 3 presents the results obtained; Section 4 concludes with a discussion of the limitations and of the research and policy implications.

## 2 Method

### 2.1 Experimental design and procedures

The general methodology used in our experiment was to leave bowls of sweets (Jelly Beans) next to the participants while they watched different videos on individual computer screens during two experimental sessions set two days apart. Sweets eating was monitored throughout the two sessions, and monetary incentives to eat or not to eat sweets were introduced during one of the videos in the first session to observe their effects on eating behaviour.

Each participant watched a total of four different videos individually, with a bowl of Jelly Beans next to them (approximately 2.2 kcal and 1.14 g per Jelly Bean). Three of the videos were in the first experimental session, while the fourth video was in the second session. During the first video, we let participants take sweets from the bowl and eat them as they pleased. We explicitly told people that they could eat sweets from the bowl as they liked. Before starting the second video, we implemented one of the three following conditions and informed participants about it:

1) *“Control” condition:* Participants could keep on eating sweets as they liked during the next video.
2) *“Eat” condition:* Participants received £3 at the end of the session if they ate at least 10 Jelly Beans during the next video.
3) *“Don't Eat” condition:* Participants received £3 if they did not eat any Jelly Beans during the next video.

Before the third and fourth videos, participants (in all the conditions) were informed again that they could eat sweets as they liked during the videos. Table 1 summarizes the structure of the different experimental conditions.

The first, third and fourth videos were approximately 10 min long and the second video approximately 5 min long. The main reason for the shorter length of the second video was that we hypothesized that 5 min would be enough to establish the incentive structure, and we wanted to avoid inducing people in the *Eat* condition to eat too many sweets, or a number of sweets that was too low for 10 min. In the other videos, 10 min provided more time to obtain good observations. All the videos were selected to be mildly boring, so that they tended to encourage sweets eating (Abramson and Stinson, 1977; Macht, 2008). The first video showed a bus journey through London filmed from inside the bus; the second video explained briefly the history of sweets in the UK (this topic was chosen to make the incentive manipulation during the second video a bit more coherent); the third video explained the bus system in London in the 1950s; the fourth video was a fragment from a documentary about animals.

After each video, the participants were asked to move to a different room where they answered a few simple questions about the content of the video and how they felt about it. Meanwhile, unbeknownst to them, the bowls of sweets were weighted with professional scales by the research assistants, and the measurements recorded to monitor eating behaviour. After answering the questions, subjects were asked go back to their computers for the next video, and also informed of any incentive that would be in place during the video.

This design was intended to allow for an analysis of the effects of the two different incentive schemes used while they were in place (during Video 2), immediately after they were removed (during Video 3), and also two days later (during Video 4). An obvious complication with the effects observed immediately after the incentives were removed is that the different amounts of sweets eaten during the video with incentives can affect subjects' appetite in the next video. Nevertheless, as the results will show, it is still possible to extract interesting insights from eating behaviour during Video 3. In addition, sweets-eating behaviour during Video 4 provides a clean test of the carryover effects of the monetary incentives used.

In the first experimental session, before starting with the first video, participants responded to various questionnaires intended to elicit additional personality and health-related information. The questionnaires included the following elements:

1) Big Five Inventory (John et al., 1991; John et al., 2008), to measure the Big Five personality dimensions (Extraversion, Agreeableness, Conscientiousness, Neuroticism, and Openness).
2) Health and Taste Attitudes Questionnaire (Roininen et al., 1999), which measures six factors that can be grouped into two main categories. The factors are: “Health Interest”, “Light Product Interest”, “Natural Product Interest”, “Craving for Sweet Foods”, “Using Food as a Reward”, and “Pleasure”. The first three factors can be grouped in the category “Healthiness” and the last three in the category “Taste”, which are intended to capture, respectively, attitudes towards the healthiness and the taste of food.
3) A question about the frequency of sweets intake.
4) A question about the desire to lose weight.
5) Two questions about height and weight to calculate the Body Mass Index of the participants.

### 2.2 Participants and experimental sessions

A total of 353 subjects participated in 35 experimental sessions. On their arrival to the lab, subjects were identified anonymously by using an ID code assigned by the online recruitment system (SONA), and they were asked to read an informed consent form and sign it if they agreed to carry on with the experiment. All subjects who came to the lab gave their consent and decided to take part in the experiment. We then asked people to draw a number at random to determine their cubicle in the lab, and participants in each session were randomly assigned to either the Control group, or the *Eat* or *Don't Eat* conditions. Randomization resulted in 133 subjects in the *Eat* condition, 112 in the *Don't Eat* condition and 108 in the *Control* condition. Attrition from the first day of the experiment to the second was 11%, so that the number of participants in the second session was 314.

The experimental protocol was approved by the LSE Research Ethics Committee, and by the Board of Directors of the Centre for the Study of Incentives in Health (CSIH), which funded the experiment. All experimental sessions were run at the LSE Behavioural Research Lab (BRL) between June and September 2012. Subjects were recruited from the volunteers in the BRL subject pool, which comprises about 5000 subjects, mostly current and former students of the University of London. There were no other eligibility or exclusion criteria to select participants. In the invitation email, subjects were not informed about the exact nature of the experiment that would be conducted. They were simply told in advance that the experiment would require coming to the lab for two separate sessions on two different days of the week (Monday and Wednesday); that each session would last about an hour; that they would receive a fixed amount of £20 for their participation in both sessions (in addition to any amount won during the sessions); and that they would have the opportunity to get an extra payment related to their tasks. Exact experimental instructions and any other materials used in the experiment are available from the authors upon request.

## 3 Results

Table 2 summarises the main results of the experiment. It contains the average number of Jelly Beans eaten, and the standard deviation, in each condition and each video. It also includes the total number of sweets eaten over the four videos, and the difference between the sweets eaten in Video 4 and in Video 1 (*Video 4-1*). Finally, Table 2 also shows the results of t-tests comparing the two incentive conditions with the control group.

As Table 2 shows, there were no significant differences in sweets-eating behaviour between the conditions during Video 1, which confirms that there were no significant sample biases. During Video 2, while the incentives were in place, the patterns obtained were exactly as expected. The *Eat* condition showed clearly the highest number of sweets eaten, followed by the *Control* condition and then the *Don't Eat* condition. All these differences are statistically significant at the 1% level: the monetary incentives to eat and not to eat were both effective in modifying behaviour in the expected direction while they were in place.

The most interesting results come from Video 3 and especially Video 4. In Video 3, right after the incentives were removed, the *Don't Eat* condition shows the lowest number of Jelly Beans eaten, followed quite closely by the *Eat* condition and then the *Control* condition. The difference between *Don't Eat* and *Control* (the two most extreme values here) is statistically significant, but the difference between *Eat* and *Control* (or between *Eat* and *Don't Eat*) is not. The most noteworthy result in Video 3 is that people ate significantly less sweets in the *Don't Eat* condition than in the *Control* condition. This result is the opposite of what would be expected on the grounds of the appetite state of the subjects: given that people in the *Don't Eat* condition ate fewer sweets during Video 2, they should have undermined their appetite less. Therefore, one would expect that subjects in the *Don't Eat* condition would eat more, not less, during the next video. Thus, this result shows a clear carryover effect of the incentives not to eat on subsequent behaviour in the same experimental session.

In Video 4, the results show that people ate considerably fewer sweets two days later in the *Don't Eat* condition than in the *Control* and the *Eat* conditions. The difference is statistically significant at 5% when comparing with the *Eat* condition and at 10% when comparing with the *Control* condition. This pattern is also clearly observed in the difference between the sweets eaten in Video 4 and in Video 1. While subjects in the *Control* and the *Eat* conditions ate more sweets in Video 4 than in Video 1, the subjects in the *Don't Eat* condition ate slightly less. In this case, the pattern is significant at 1% when comparing with the *Eat* condition and at 5% when comparing with the *Control* condition. This result shows a clear carryover effect of the incentives not to eat, which affected sweets-eating behaviour two days after the incentives were removed. The result is particularly striking given that participants were only exposed to the monetary incentives for 5 min two days before. On the contrary, the behaviour of people in the *Eat* condition was indistinguishable from the *Control* condition two days later.

To complement these patterns, Table 3 shows the results of a formal regression analysis. In a first set of regressions (*Models 1–5*), we stack the four videos together and treat the data as a panel with four time observations per subject. We use pooled panel (population-averaged) Ordinary Least Squares (OLS) regressions with robust standard errors clustered at the subject level to account for individual heterogeneity, and for the fact that error terms can be potentially correlated across videos for the same subject. The dependent variable in all the pooled OLS regressions is the number of Jelly Beans eaten during the different videos, while the explanatory variables in the various specifications include: dummies for the *Eat* and *Don't Eat* conditions; dummies for *Video2*, *Video3*, and *Video4*; treatment–video interaction terms (e.g., Eat*V2 is the interaction term for the sweets eaten during Video 2 in the *Eat* condition); scores on the five personality dimensions (*PersE*, *PersA*, *PersC*, *PersN*, and *PersO*, which stand for Extraversion, Agreeableness, Conscientiousness, Neuroticism, and Openness, respectively); scores for the two main categories of the Health and Taste Attitudes Questionnaire (HTAQ) (*Healthiness* and *Taste*, increasing with the degree of concern for the healthiness and the taste of food, respectively); self-reported frequency of sweets intake (*SweetsIntake*); self-declared intention to lose weight (*LoseWeight*); and calculated scores for the Body Mass Index (*BMI*).

The final regression (*Model 6*) uses the number of Jelly Beans eaten during Video 1 as the dependent variable, and it is simply meant to give a sense of how the personality and health-related variables affected sweets eating in the absence of any effects of monetary incentives. The explanatory variables are the same as above, with the exception of the treatment dummies (and of course of the video dummies and interaction terms). Estimates in this case are obtained using a standard OLS regression adjusting the variance-covariance matrix for possible heteroskedasticity.

The results of the baseline pooled panel OLS regression (*Model 1*) confirm significant and negative effects of the *Don't Eat* interaction terms: subjects assigned to the incentives not to eat, ate significantly fewer Jelly Beans than participants in the control group, not only during Video 2 but also in Videos 3 and 4. On the other hand, there are no significant interaction effects for the subjects in the *Eat* condition, apart from the obvious one for Video 2. Finally, while the main treatment dummies are not significant, the effects of the time dummies are as expected: compared to the first video, subjects across all conditions tended to eat fewer Jelly Beans in Video 2 (which was only 5 min long) and in Video 3, and more in Video 4 (two days later).

When the scores on the five personality dimensions are added as controls in the pooled OLS regression (*Model 2*), the estimates show significant and positive effects of *Agreeableness* and *Neuroticism*, and a significant negative effect for *Openness*. Importantly, the previously described effects of the *Don't Eat* interaction terms are fully confirmed: subjects previously paid not to eat continued eating significantly less in Videos 3 and 4.

The effects of these interaction terms are also robust to using as alternative controls the variables for the HTAQ dimensions (*Model 3*), self-reported frequency of sweets intake, self-declared intention to lose weight, and individual BMI (*Model 4*). Curiously, self-reported concern for the *Healthiness* of food had a positive effect on sweets eating. Frequency of sweets intake and BMI had also positive effects, and desire to lose weight a negative one: subjects with higher BMI and who usually eat more sweets also ate more sweets in the experiment, while people who had a desire to lose weight ate less.

Results are qualitatively unaltered when all control variables are introduced simultaneously in the pooled OLS regression (*Model 5*): subjects assigned to the *Don't Eat* condition continue eating significantly less than in the control group in Videos 3 and 4 (although in the latter video the effect is only significant at a 10% level).

It is important to note that the results of the pooled OLS regressions reported above are broadly confirmed when using random effects panel models; when all variables are standardized to minimize multicollinearity issues potentially arising from the interaction terms; and when regressions are replicated excluding the three subjects in the sample who suffered from diabetes (these additional analyses are available from the authors on request). The results of the final OLS regression (*Model 6*) show that the main personality influence on sweets eating was *Neuroticism*, which might suggest that people who tended to get more anxious during the videos ate more sweets. Also *Openness* seems to have had some influence on eating behaviour, with more open people eating fewer sweets, but the effect is only significant at the 10% level. The regression also confirms the positive effects described above for the self-reported concern for the *Healthiness* of food, frequency of sweets intake, and BMI, and the negative effect of desire to lose weight. Also these results are confirmed when the OLS regression is replicated excluding the three subjects in the sample who suffered from diabetes (analysis also available on request).

Finally, we conduct a split-sample analysis to explore under which conditions the different incentives affect behaviour, and in particular whether the financial incentives induced consumption in the ‘under-eaters’ and reduced consumption in the ‘over-eaters’. This split sample analysis is important for both research and policy purposes, because one might worry that inducing consumption in ‘under-eaters’ may produce over-eating in people who were already eating more or that reducing consumption in ‘over-eaters’ may result in under-consumption in people who were consuming appropriately in the absence of incentives.

In Table 4, we present additional regressions splitting the sample in two sub-groups, according to whether the initial sweets consumption in Video 1 was below or above the median consumption. The results for the two subsamples above (*Model 1a*) and below (*Model 1b*) the median consumption, are reported in Table 4, together with the analogous regression for the whole sample for the sake of comparison (*Model 1*). The findings are of key interest as they confirm that incentives not to eat primarily had a lasting effect in the cases where a change in the target behaviour is envisaged the most, namely the ‘over-eaters’ who ate sweets above the median level.

First, as in the whole sample, both incentives to eat and not to eat were effective in changing the eating behaviour of subjects who in Video 1 ate sweets below the median level. For subjects who in Video 1 already ate sweets above the median level, only incentives not to eat were effective in changing eating behaviour. Second, like in the whole sample, none of the sub-groups exhibited significant carryover effects of monetary incentives in the *Eat* condition. Furthermore, the carryover effects of the incentives not to eat seem limited to subjects who in Video 1 ate sweets above the median level. These subjects (but not the ones who ate sweets below the median level) continued eating significantly fewer sweets in Videos 3 and 4.

## 4 Conclusions

We have investigated the effects of two different types of monetary incentives on sweets-eating behaviour: incentives to eat and incentives not to eat. These two incentives schemes have been widely used in research and policy but they have never been directly compared head-to-head using the same target behaviour and the same environment. We found that both types of incentives were effective in modifying behaviour as expected while they were in place, but only incentives not to eat had significant carryover effects on eating. People who were paid not to eat sweets for only 5 min, showed reduced sweets eating two days after this incentive was removed.

One interpretation of these results is that paying people not to eat helped them to exert and build up their self-control. Then this improved ability to exert self-control and refrain from engaging in a behaviour that may be undesirable carried over to subsequent situations where incentives were absent. On the contrary, paying people to eat was not so successful in building-up a habit that carried over to future contexts.

An alternative explanation may be that paying people not to eat sweets simply primed them with the notion that not eating sweets is something good and influenced subsequent behaviour, while incentives to eat failed to successfully prime people with the idea that eating sweets is good. This is consistent with the well documented ‘bad is stronger than good’ effect, which is the notion that negative messages are more salient and easier to retain than positive ones (Baumeister et al., 2001). Similar effects have also been documented in the context of nutritional food labelling, where people seem to react more strongly to negative health messages than to positive ones (Fox et al., 2002).

We also conducted a split-sample analysis to look at whether the effects of the different types of incentives induced consumption in the ‘under-eaters’ and reduced consumption in the ‘over-eaters’, for whom a behavioural change can be desired the most in practical health-policy applications. The split-sample analysis reassuringly confirms that incentives not to eat primarily had a lasting effect on the subjects who ate sweets above the median level. This strengthens the policy implications, as it indicates that the effects of monetary incentives to improve poor behaviour do not appear to have adverse spillover effects on the subjects who are not directly targeted by the policy.

While our results pertain to a specific context and should not be interpreted as general evidence against the carryover effects of incentives to act, they suggest that incentives to abstain from acting are likely to have more powerful and long-lasting effects on behaviour, at least in circumstances that have similar features to our set-up. What characterizes our set-up is mainly an ambivalent health situation where subjects can choose to act in a way that is pleasurable and tempting, but potentially harmful or unwanted at a deeper level. The same pattern applies to a large number of health behaviours of high policy relevance, like alcohol drinking and unsafe sex, which makes our results relevant to these applied areas.

It is worth noting, however, that another relevant category of health-related situations is that in which people can choose to act in a way that is beneficial or desirable, but also costly and effortful, so that they are tempted to abstain from acting. Such situations could include behaviours like exercising, eating vegetables, taking a screening test or vaccination, or going to see the doctor. These situations are essentially the opposite of our set-up in the sense that the behaviour that requires self-control is to act instead of to abstain from acting. In these cases, incentives to act and not to act are likely to have different consequences.

Based on our results, we would expect that the patterns produced by incentives to act and to abstain from acting are likely to reverse, so that incentives to act may have stronger carryover effects than incentives to refrain from acting. Further research along these lines is needed to fully uncover the effects of different types of incentives in these different circumstances.

## Figures and Tables

**Table 1:** Structure of the different experimental conditions.

	Session 1			Session 2
	Video 1: 10 min	Video 2: 5 min	Video 3: 10 min	Video 4: 10 min
Eat Condition	No incentive	**Incentive to eat**	No incentive	No incentive
Don't Eat Condition	No incentive	**Incentive not to eat**	No incentive	No incentive
Control Condition	No incentive	No incentive	No incentive	No incentive

**Table 2:** Average number of Jelly Beans eaten per video and condition.

	Video 1	Video 2	Video 3	Video 4	TOTAL	Video 4-1
Eat Condition	16.89 (24.86)	14.62*** (13.56)	4.13 (16.29)	24.03 (33.73)	61.59 (64.59)	7.34 (21.86)
Don't Eat Condition	16.98 (23.86)	0.04*** (0.70)	3.09*** (9.48)	16.47* (20.85)	36.22** (45.71)	−0.18** (20.31)
Control Condition	15.66 (20.77)	5.88 (8.46)	6.92 (11.31)	23.14 (32.10)	52.88 (67.28)	6.92 (20.14)
OVERALL	16.54 (23.30)	7.32 (11.33)	4.64 (13.01)	21.43 (29.68)	50.86 (60.89)	4.82 (21.07)

Notes: Standard deviations in parentheses. Results of t-tests comparing the incentive conditions to the control group: *p < .10, **p < .05, ***p < .01.

**Table 3:** Regression analysis for sweets eaten during the videos.

Sweets eating	Model 1	Model 2	Model 3	Model 4	Model 5	Model 6
Eat	1.229 (2.942)	0.765 (2.950)	2.357 (3.224)	1.854 (2.885)	3.161 (3.084)	
Don't eat	1.324 (3.015)	0.051 (3.102)	1.029 (3.236)	1.841 (2.975)	1.092 (3.132)	
Video 2	−9.777*** (1.516)	−9.859*** (1.532)	−10.23*** (1.606)	−9.859*** (1.530)	−10.23*** (1.612)	
Video 3	−8.741*** (1.405)	−8.802*** (1.414)	−8.936*** (1.492)	−8.881*** (1.416)	−8.961*** (1.491)	
Video 4	7.632*** (2.100)	7.276*** (2.073)	7.592*** (2.162)	7.336*** (2.069)	7.218*** (2.122)	
Eat*V2	7.507*** (2.635)	7.683*** (2.678)	7.135** (2.896)	7.683*** (2.676)	7.135** (2.906)	
Eat*V3	−4.018 (3.234)	−3.913 (3.289)	−5.235 (3.586)	−3.834 (3.289)	−5.211 (3.593)	
Eat*V4	−0.494 (3.250)	−0.435 (3.268)	−1.179 (3.442)	−0.436 (3.277)	−0.839 (3.391)	
Don't Eat*V2	−7.168*** (2.718)	−6.934** (2.784)	−6.884** (2.958)	−6.934** (2.782)	−6.884** (2.968)	
Don't Eat*V3	−5.151** (2.402)	−4.852** (2.446)	−4.656* (2.582)	−4.773* (2.446)	−4.633* (2.585)	
Don't Eat*V4	−8.145*** (2.930)	−7.832*** (2.933)	−8.247*** (3.156)	−7.786*** (2.938)	−7.948** (3.120)	
PersE		−1.059 (2.524)			−1.916 (2.812)	−3.781 (4.090)
PersA		5.306** (2.944)			5.865* (3.089)	2.893 (4.404)
PersC		3.488 (3.494)			3.976 (3.599)	5.172 (4.826)
PersN		5.981** (2.348)			4.946** (2.318)	7.307** (3.145)
PersO		−4.702* (2.581)			−5.738** (2.765)	−7.156* (3.962)
Healthiness			0.219* (0.118)		0.221** (0.112)	0.433*** (0.161)
Taste			0.014 (0.093)		0.047 (0.084)	0.035 (0.124)
SweetsIntake				1.813*** (0.611)	1.921*** (0.689)	3.615*** (1.145)
LoseWeight				−0.828** (0.419)	−0.698 (0.454)	−1.369** (0.674)
BMI				0.907*** (0.309)	0.955*** (0.312)	1.357*** (0.476)
Constant	15.66*** (1.999)	−11.18 (14.49)	−2.562 (11.35)	0.236 (6.765)	−39.84* (23.02)	−49.65 (31.26)
Observations	1372	1216	1216	1339	1216	312
R-squared	0.126	0.185	0.129	0.153	0.185	0.113

Notes: *, ** and *** indicate significance at the level of 10%, 5% and 1% respectively. Models 1–5 are pooled panel OLS regressions with SE clustered at subject level. Model 6 is heteroskedasticity-robust OLS regression for Video 1.

**Table 4:** Regression analysis for sweets eaten during the videos: split sub-samples.

Sweets eating	Above median	Below median	Whole sample
	Model 1a	Model 1b	Model 1
Eat	1.738 (4.641)	−0.418 (0.531)	1.229 (2.942)
Don't eat	3.266 (4.708)	−0.565 (0.531)	1.324 (3.015)
Video 2	−19.15*** (2.465)	−0.745 (0.481)	−9.777*** (1.516)
Video 3	−17.75*** (2.145)	−0.137 (0.791)	−8.741*** (1.405)
Video 4	7.674** (3.608)	6.545*** (1.968)	7.632*** (2.100)
Eat*V2	6.166 (4.152)	9.684*** (1.955)	7.507*** (2.635)
Eat*V3	−7.186 (5.629)	0.121 (1.381)	−4.018 (3.234)
Eat*V4	2.153 (5.710)	−3.272 (2.246)	−0.494 (3.250)
Don't Eat*V2	−13.21*** (4.316)	−1.325** (0.581)	−7.168*** (2.718)
Don't Eat*V3	−8.772** (3.813)	−1.565* (0.871)	−5.151** (2.402)
Don't Eat*V4	−12.17** (5.312)	−2.290 (2.323)	−8.145*** (2.930)
Constant	29.18*** (3.108)	2.618*** (0.422)	15.66*** (1.999)
Observations	686	686	1372
R-squared	0.246	0.161	0.126

Notes: *, ** and *** indicate significance at the level of 10%, 5% and 1% respectively. All models are pooled panel OLS regressions with SE clustered at subject level.
